# Microencapsulated Insulin-Like Growth Factor-1 therapy improves cardiac function and reduces fibrosis in a porcine acute myocardial infarction model

**DOI:** 10.1038/s41598-020-64097-y

**Published:** 2020-04-28

**Authors:** Claudia Báez-Díaz, Virginia Blanco-Blázquez, Francisco-Miguel Sánchez-Margallo, Antoni Bayes-Genis, Irene González, Ana Abad, Rob Steendam, Okke Franssen, Itziar Palacios, Belén Sánchez, Carolina Gálvez-Montón, Verónica Crisóstomo

**Affiliations:** 1 Jesús Usón Minimally Invasive Surgery Centre, Cáceres, Spain; 2 CIBERCV, Madrid, Spain; 3 ICREC (Heart Failure and Cardiac Regeneration) Research Programme, Health Sciences Research Institute Germans Trias i Pujol (IGTP), Badalona, Barcelona Spain; 4 Innocore Pharmaceuticals, Groningen, The Netherlands; 5 Nanomi BV, Oldenzaal, The Netherlands; 6 Tigenix, Madrid, Spain

**Keywords:** Interventional cardiology, Preclinical research

## Abstract

Insulin-like growth factor-1 (IGF-1) has demonstrated beneficial effects after myocardial infarction (MI). Microencapsulation of IGF-1 could potentially improve results. We aimed to test the effect of an intracoronary (IC) infusion of microencapsulated IGF-1 in a swine acute MI model. For that purpose IC injection of a 10 ml solution of 5 × 10^6^ IGF-1 loaded microspheres (MSPs) (n = 8, IGF-1 MSPs), 5 × 10^6^ unloaded MSPs (n = 9; MSPs) or saline (n = 7; CON) was performed 48 hours post-MI. Left ventricular ejection fraction (LVEF), indexed ventricular volumes and infarct size (IS) were determined by cardiac magnetic resonance at pre-injection and 10 weeks. Animals were euthanized at 10 weeks, and myocardial fibrosis and vascular density were analysed. End-study LVEF was significantly greater in IGF-1 MSPs compared to MSPs and CON, while ventricular volumes exhibited no significant differences between groups. IS decreased over time in all groups. Collagen volume fraction on the infarct area was significantly reduced in IGF-1 MSPs compared to CON and MSPs. Vascular density analysis of infarct and border zones showed no significant differences between groups. In conclusion, the IC injection of 5 × 10^6^ IGF-1 loaded MSPs in a porcine acute MI model successfully improves cardiac function and limits myocardial fibrosis, which could be clinically relevant.

## Introduction

Cardiovascular diseases, especially ischemic heart disease, are the leading cause of mortality worldwide accounting for almost 4 million deaths a year in Europe^1,2^. Conventional treatments such as angioplasty and coronary stenting have contributed to reduce early mortality after an acute myocardial infarction (MI)^3^. However, such therapies are only palliative and do not recover the damaged myocardial tissue^4^, so that these diseases still represent a major unmet medical need.

In the last two decades stem cell therapy has become a promising treatment option for ischemic cardiomyopathy^5^. As a result, the administration of various cell types has been proposed to address this problem but has shown only moderate improvements in cardiac function^6^.

Recently, several studies suggest that the beneficial effect of stem cells does not lie in their multiplication, but in their paracrine actions^7^. Based on this insight, current research directions in regenerative cardiology are moving to a cell-less approach, since it is known now that stem cells are able to secrete combinations of biomolecules that modulate the composition of the damaged cardiac environment contributing to functional tissue repair by stimulating the migration, proliferation and survival of endogenous cardiac progenitor cells (eCSCs)^8,9^, as well as attenuating fibrosis and modulating inflammation^10,11^.

Among the secreted substances, there are different cytokines, extracellular vesicles and growth factors including insulin-like growth factor-1 (IGF-1), hepatocyte growth factor (HGF), angiopoietin 2 or vascular endothelial growth factors (VEGF) that seem to limit myocardial inflammation and post-infarct scar^12,13^. We focused our work on IGF-1, since there are different studies that have demonstrated a limitation on reperfusion damage via prosurvival and antiapoptotic effects on cardiac cells, as well as angiogenic effects on endothelial cells^14,15^. Besides, microencapsulated drug delivery allows for sustained release over a specific time that could improve results with a single administration procedure.

Accordingly, the goal of the present study was to assess the safety and effectiveness of an intracoronary (IC) infusion of microencapsulated IGF-1 after acute MI in a clinically relevant swine model of reperfused MI.

## Methods

### Experimental protocol

The study protocol was approved by the Institutional Animal Care and Use Committee, and it complied fully with the Directive 2010/63/EU of the European Parliament on the protection of animals used for scientific purposes.

Young female Large White swine weighing 30–35 kg were used for this study (n = 27). After a complete physical examination, healthy pigs were included in the protocol. Animals received oral antiarrhythmics and antithrombotics before model induction continuing after it as described in Table 1.

**Table 1:** Antiarrhythmic and antithrombotic medication administered during the study.

Drug	Posology
Amiodarone	400 mg from 5 days prior to infarction to 3 days after it
Acetylsalicylic acid	500 mg from 24 hours before model induction continuing until euthanasia
Clopidogrel	300 mg 24 hours before model induction; 75 mg until euthanasia

### Anaesthesia and analgesia

After a fasting period of 24 hours, premedication of the animals was achieved by an intramuscular (IM) injection of ketamine (20 mg/kg). Ten minutes later, midazolam (0.2 mg/kg) and buprenorphine (10 µg/kg) were injected intravenously (IV). Anaesthesia was induced with IV etomidate (0.5–1 mg/kg). After endotracheal intubation, anaesthesia was maintained using inhaled sevoflorane (1.8–2% inspiratory fraction) combined with a continuous IV infusion of 2% lidocaine and midazolam (0.05 mg/kg/h), providing a previous IV bolus of lidocaine (2 mg/kg). Endotracheal tubes were connected to a semi closed circular anaesthetic circuit attached to a ventilator (Maquet Flow i) with a fresh gas flow rate of 1 l/min (0.4/0.6 mixture of oxygen and air). Controlled ventilation was established with a tidal volume of 10 ml/kg to obtain normocapnia (with a CO_2_ pressure of 40–45 mmHg)^16^.

Adequate postoperative analgesia was ensured by IM buprenorphine (10 μg/kg/12 h) during the first 24 hours followed by placement of a fentanyl transdermic release patch (25 μg/h).

### Infarct induction

Infarct creation was accomplished as previously described^16^. Briefly, anesthetized swine were fixed at the table in the dorsal decubitus with caudal extension of the hind limbs. Heparin (150 IU/kg) was injected IV 5 minutes before percutaneous insertion of a 7 Fr introducer sheath (Terumo, Inc. Tokyo, Japan) into the femoral artery. Fluoroscopic guidance (Philips Mobile Digital Angiographic System-BV Pulsera, Philips Medical Systems, Best, The Netherlands) was used to place a 6 Fr hockey stick guiding catheter (Mach 1, Boston Scientific Corporation, Natick, MA, USA) at the origin of the left coronary artery and advance a 0.014” guidewire (Hi-torque. Abbott Vascular, Santa Clara, CA, USA) inside the left anterior descending artery (LAD). In order to prevent coronary spasm 150 μg of nitroglycerin were administered through the catheter. Subsequently, a coronary balloon (Xperience iVascular, Barcelona, Spain) of appropriate diameter was advanced over the guidewire and inflated immediately below the origin of the first diagonal branch of the LAD occluding this artery for 90 minutes. Contrast injections were performed through the guiding catheter after balloon inflation and before deflation to assess correct occlusion.

Episodes of ventricular fibrillation during the animal model induction were treated by manual chest compressions and 200 J biphasic defibrillation shocks (Zoll M series biphasic 200 J, Zoll Medical Corporation, Massachusetts, USA) as well as pharmacological therapy when needed. After balloon deflation, coronary flow was checked by manual contrast injection and scored following the Thrombolysis In Myocardial Infarction (TIMI) grade flow. Anaesthesia was maintained for another hour and pigs (once recovered) were carried to the animal housing facility. All animals received prophylactic antibiotics for 5 days after infarct induction.

### IGF-1 loaded microspheres preparation

Biodegradable microspheres (MSPs) composed of proprietary SynBiosys multi-block co-polymers (Innocore Pharmaceuticals) and manufactured to a predetermined particle size adequate for IC administration (16 µm) with the proprietary Microsieve technology (Nanomi) were loaded with 5% IGF-1. Growth factor loading was 95 µg per 10^6^ MSPs, which were slowly released over 3 weeks (90% release completed by the end of week 3). To prepare the microspheres, IGF-1 was purified from commercially available mecasermin (Increlex 40 mg/4 ml, Ipsen Pharma) which contains recombinant DNA-engineered human insulin-like growth factor-1 (rhIGF-1) and processed (washed and concentrated) to remove excipients (such as bencyl alcohol) and concentrate the protein to allow for correct IGF-1 loading of the microspheres. Thus, a concentration of IGF-1 up to 95 mg/ml in the primary Synbiosis polymers emulsion was obtained, which considering a target IGF-1 loading of 5 wt% yielded the aforementioned dose of 95 µg per 10^6^ MSPs. For the injection procedure, MSPs concentration was adjusted to 0.5 × 10^6^ MSPs/ml.

### Group allocation and IC infusion

Animals were allocated to control (saline injection, CON), blank (5 million unloaded MSPs injection, MSPs) or IGF-1 (5 million IGF-1 loaded MSPs, IGF-1 MSPs) group before infarct induction. In the three groups the infusion was performed blindly 48 hours after infarction, immediately after acquiring a Cardiac Magnetic Resonance (CMR) study. Access to the LAD was established using the same protocol described for infarct creation. For injection a 3 Fr microcatheter was used (Microferret infusion catheter, Cook Medical. Bloomington, IN, USA) at an injection rate of 1 ml/minute. Nitroglycerin was administered via the microcatheter prior to the beginning of the injection (400 µg) and a coronary angiogram obtained. The total volume of 10 ml was divided in four injection cycles separated by 3 minutes rest periods. Once the stated volume was infused, we waited for 5 minutes before obtaining a coronary angiogram to assess coronary TIMI grade flow again. The femoral sheath was then removed and haemostasia of the puncture site was achieved by manual compression.

Blood samples were taken for cardiac troponin I (cTnI) assay at baseline and after reperfusion, immediately before and 2 hours after IC infusion as well as before euthanasia at 10 weeks (AQT90 Flex, Radiometer Iberica SL, Madrid, Spain).

### CMR examinations

CMR studies were performed before injection as well as 10 weeks after it. For that purpose, swine were accommodated inside the MR system (Intera 1.5 T, Philips Medical Systems. Best, The Netherlands) in the sternal decubitus and images were obtained in the intrinsic cardiac planes (short axis, vertical long axis and horizontal long axis views). Left ventricular function was measured on breath hold gradient echo cine images that were acquired over the complete left ventricle (LV). As previously described^16^, typical parameters used were: slice thickness: 8 mm, no gap, Field of view (FOV): 320x 320 x 80, matrix: 192x 192, flip angle: 60°, repetition time/echo time (TR/TE): 4.4/2.2. Measurement of infarct size (IS) was performed on short axis images that were acquired 10 minutes after the administration of a gadolinium-based contrast agent (0.2 mmol/kg) and making use of a breath-hold 3D gradient-echo inversion-recovery sequence. A Look-Locker sequence was used to choose the inversion time for each sequence, selecting the time that provided the best nulling of the myocardial signal. Typical parameters used were slice thickness: 8 mm, no gap, FOV: 330 x 330 x 50, matrix: 224 × 200, flip angle: 15°, TR/TE: 4.9/1.67. CMR analysis was carried out by a researcher blinded to the group allocation. Left ventricular ejection fraction (LVEF), end diastolic volume (EDV) and end systolic volume (ESV) were calculated by defining endocardial and epicardial borders in end diastolic and end systolic short axis images, in all slices. Since the animals used in the present study were still growing, in order to minimize the influence of weight gain on our interpretation of the results, EDV and ESV were indexed to Body Surface Area (EDVi and ESVi)^16^. Delayed enhancement images were used to calculate IS which was achieved by specifying the healthy and infarcted areas to obtain the percentage of infarcted LV.

### End of study and post-mortem examinations

Ten weeks after infarct creation (immediately after the second CMR study) TIMI grade flow scoring system was used to evaluate coronary flow. After that, euthanasia was carried out by a lethal dose of potassium chloride (1–2 mmol/kg) while under deep anaesthesia. Hearts were explanted and cut into 0.5 cm thick slices. One section was maintained in a 1% solution of 2,5,3-triphenyl tetrazolium chloride (TTC) in phosphate buffer at 37 °C for 10 minutes and photographed. In addition, samples were taken from the infarct, border and remote areas for pathological analysis, embedded in paraffin, sliced into 5 μm thick sections and stained with Haematoxylin-eosin (H/E) and Masson’s trichrome (MT)^16^. Picrosirius red staining was performed to analyse myocardial fibrosis. The collagen volume fraction (CVF), collagen I, collagen III, and collagen I/III ratio were measured in the infarct core and remote zones. Likewise, biopsies from healthy myocardium, border and infarct zones were obtained, embedded in Tissue-Tek O.C.T compound and snap-frozen in methylbutane, and stored until analysis. Vessel density was analysed in 10μm frozen sections with biotinylated Griffonia simplicifolia lectin I B4 (IsoB4; 1:50; Vector Labs, Burlingame, CA, USA) and Streptavidin-Alexa488 (1:500; Invitrogen) immunostaining.

### Statistical analysis

Data are presented as means ± standard deviations. Data were checked for normality using the Shapiro Wilk test. Differences between groups were identified and compared using the Kruskall-Wallis and Mann-Whitney U tests (not normally distributed variables) or One-way ANOVA and Student´s T-Test (normally distributed variables).

Values of p < 0.05 were considered significant. Calculations were performed using the SPSS 18.0 statistical package for Windows (SPSS Inc, Chicago, Ill).

## Results

### Infarct induction and IC infusion

Three animals died during infarct induction, which resulted in the following distribution: CON (n = 7), MSPs (n = 9) and IGF-1 MSPs (n = 8). No further animal deaths, of cardiac or any other origin, were seen prior to IC therapy. In MSPs one animal died during the second CMR examination and another one during IC delivery. In the remaining swine injection was completed in absence of major cardiac events.

Infarction was successfully induced in all surviving animals, as demonstrated by a significant increase in cTnI values in all animals 2 hours after model creation (Table 2) (CON and MSPs: p = 0.007; IGF-1 MSPs: p = 0.001; t test for related samples). After infarction, cTnI was significantly higher in IGF-1 MSPs than in CON (p = 0.009) and MSPs (p = 0.019) (One-way ANOVA, post-hoc Bonferroni test). A slight decrease in cTnI levels was seen in the three groups after therapy administration that was not significant in any case. Ten weeks after infarct induction, cTnI returned to clinically normal ranges.

**Table 2:** Cardiac TnI values (μg/l) measured during the study.

Group	Baseline	Reperfusion	Pre-injection	Post-injection	End study
CON	0.016 ± 0.009^a^	9.086 ± 6.067*^,a^	11.657 ± 6.445	10.571 ± 5.110	0.012 ± 0.004
MSPs	0.026 ± 0.027^a^	11.878 ± 9.868**^,a^	12.256 ± 11.398	7.713 ± 3.601	0.029 ± 0.045
IGF-1 MSPs	0.043 ± 0.049^a^	27.000 ± 13.126*^,^**^,a^	9.813 ± 4.475	9.138 ± 4.429	0.012 ± 0.005

Data presented as mean±standard deviation. Intragroup and Intergroup comparisons at each time point are denoted by ^a^ p < 0.05 and * p < 0.05, respectively.

Pre-injection coronary flow is detailed in Table 3. Although no increases in cTnI values were detected after injection, there was a worsening in coronary flow in 4 animals from the MSPs and the IGF-1 MSPs groups. At 10 weeks TIMI flow grade scoring demonstrated TIMI 3 in 6 swine from CON and MSPs, as well as 7 pigs from IGF-1 MSPs. All remaining animals showed TIMI 2 at the end of the study (except 2 animals from MSPs group, which died before final TIMI flow evaluation).

**Table 3:** Evolution of coronary flow determined by TIMI Grade Flow scoring system.

Group	Pre-injection TIMI flow	Post-injection TIMI flow	End-study TIMI flow
CON	TIMI 3 (n = 2) TIMI 2 (n = 5)	TIMI 3 (n = 2) TIMI 2 (n = 5)	TIMI 3 (n = 6) TIMI 2 (n = 1)
MSPs	TIMI 3 (n = 6) TIMI 2 (n = 3)	TIMI 3 (n = 2) TIMI 2 (n = 6) n/a (n = 1)	TIMI 3 (n = 6) TIMI 2 (n = 1) n/a (n = 2)
IGF-1 MSPs	TIMI 3 (n = 8)	TIMI 3 (n = 4) TIMI 2 (n = 2) TIMI 1 (n = 2)	TIMI 3 (n = 7) TIMI 2 (n = 1)

### CMR studies

**Figure 1:**
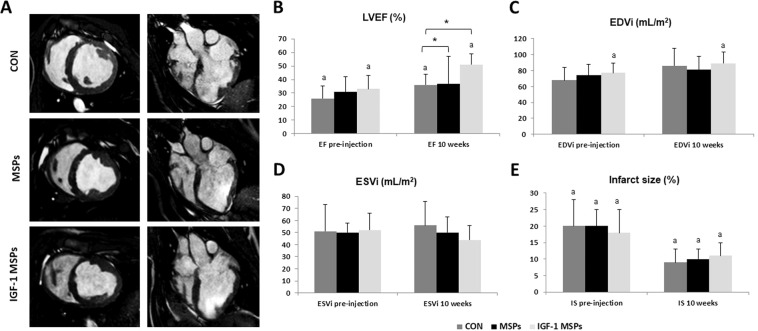
CMR results (**A**) Representative CMR cine images in short axis (left) and four chamber views (right) of the different groups. (**B**) Evolution of LVEF through the study in CON (n = 7), MSPs (n = 7) and IGF-1 MSPs (n = 8). LVEF increased significantly over time in CON and IGF-1 MSPs (^a^ p < 0.05). At 10 weeks LVEF was significantly greater in IGF-1 MSPs compared to CON and MSPs (* p < 0.05). (**C**) Evolution of EDVi through the study in CON (n = 7), MSPs (n = 7) and IGF-1 MSPs (n = 8). EDVi (ml/m^2^) increased significantly over time in IGF-1 MSPs (^a^ p < 0.05). No significant differences between groups were seen. (**D**) Evolution of ESVi through the study in CON (n = 7), MSPs (n = 7) and IGF-1 MSPs (n = 8). No significant differences over time nor between groups were observed in ESVi (ml/m^2^). (**E**) Evolution of IS through the study in CON (n = 7), MSPs (n = 7) and IGF-1 MSPs (n = 8). Significant decreases over time were seen in all groups in IS (^a^ p < 0.05). No significant differences between groups were detected in this parameter.

Cardiac function parameters are shown in Table 4. No statistically significant differences were seen between groups prior to IC injection. LVEF increased over time in all groups, significantly so in CON (p = 0.031; T test for related samples) and IGF-1 MSPs (p = 0.001; T test for related samples). At 10 weeks, LVEF was significantly different between groups (p = 0.022, Kruskall-Wallis), due to IGF-1 MSPs being significantly greater than MSPs (p = 0.049, Mann-Whitney U test) and CON (p = 0.008, Mann-Whitney U test) (Fig. 1B).

**Table 4:** Cardiac parameters calculated from CMR exams performed through the study. Data presented as mean±standard deviation.

Group	CON	MSPs	IGF-1 MSPs
LVEF (%) pre-injection	26 ± 9^a^	31 ± 11	33 ± 10^a^
LVEF (%) 10 weeks	36 ± 8^a,^*	37 ± 20**	51 ± 8^a,^*^,^**
EDVi (ml/m^2^) pre-injection	68 ± 16	74 ± 14	77 ± 12^a^
EDVi (ml/m^2^) 10 weeks	86 ± 22	81 ± 17	89 ± 14^a^
ESVi (ml/m^2^) pre-injection	51 ± 22	50 ± 8	52 ± 14
ESVi (ml/m^2^) 10 weeks	56 ± 20	50 ± 13	44 ± 12
IS (%) pre-injection	20 ± 8^a^	20 ± 5^a^	18 ± 7^a^
IS (%) 10 weeks	9 ± 4^a^	10 ± 3^a^	11 ± 4^a^

Intragroup and Intergroup comparisons at each time point are denoted by ^a^ p < 0.05 and *p < 0.05, respectively.

Regarding indexed ventricular volumes, EDVi increased in the three study groups over time, significantly so in IGF-1 MSPs (p = 0.023; T test for related samples). However, no statistical significance was reached between the three groups (Fig. 1C). Similarly, ESVi increased slightly over the 10 weeks period in CON group, while it remained stable in MSPs and decreased in IGF-1 MSPs. At the end of the study, a clear trend towards decreased ESVi was seen in IGF-1 MSPs (Fig. 1D).

IS decreased significantly over time in all groups (CON: p = 0.002; MSPs: p = 0.007 and IGF-1 MSPs: p = 0.003 (T test for related samples) in absence of significant differences between them (Fig. 1E). A greater thinning of the septal ventricular wall was visible in the CON and MSPs group, while this wall thinning was less evident in IGF-1 MSPs as shown in Fig. 1A.

### End of study and post-mortem examinations

**Figure 2:**
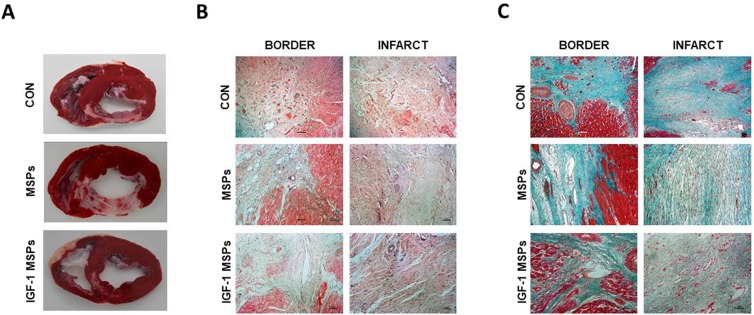
Macroscopical and histological appearance of the infarcts. (**A**) TTC staining shows the extension of infarcted tissue in the different groups. (**B**) Representative images from the three study groups of border and infarct zones after H/E staining. (**C**) Representative images from the three study groups of border and infarct zones after MT staining.

After euthanasia, TTC stained heart slices showed transmural fibrous scars of varied extension and anteroseptal location in all samples (Fig. 2A). As previously seen in CMR studies wall thinning was more evident in animals belonging to the CON and MSPs groups. In the IGF-1 MSPs group, thinning was less, and the scars of these hearts were composed of a mixture of infarcted and viable tissue.

H/E and MT staining showed no evident anatomopathological differences between CON (n = 6), MSPs (n = 6), and IGF-1 MSPs (n = 6) groups (Supplementary Fig. S1) neither in remote, nor in infarct and border zones regarding viable tissue presence (Fig. 2B,C).

**Figure 3:**
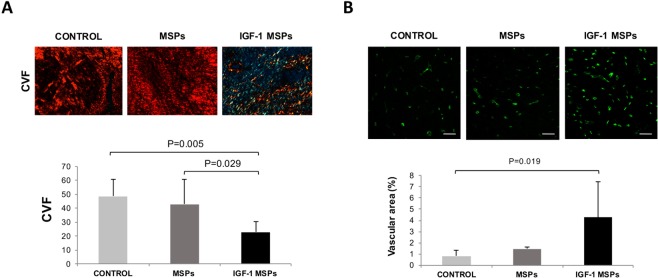
(**A**) Microphotographs of Picrosirius red staining under polarized light optical microscopy showing the CVF differences in myocardial scar among CON (n = 7), MSPs (n = 7) and IGF-1 MSPs (n = 7) groups. At the bottom of panel A histogram of the CVF analysis in infarct zone in CON (n = 7), MSPs (n = 7) and IGF-1 MSPs (n = 7). CVF was significantly lower in IGF-1 MSPs compared to CON and MSPs (p = 0.005 and p = 0.029, respectively; Tukey HSD Test). Scale bar = 50 μm. (**B**) Vascular area analysis. Representative images from CON (n = 7), MSPs (n = 6) and IGF-1 MSPs (n = 8) groups of remote zones after Isolectin B4 (green) immunostaining. At the bottom, histogram of the vascular area (%) analysis in remote zone of the three study groups. Vessel density in remote myocardium was significantly higher in IGF-1 MSPs in comparison to CON (p = 0.019; Tukey HSD Test). Scale bar = 50 μm.

Picrosirius red staining confirmed that there were no differences between CON (n = 7), MSPs (n = 7) and IGF-1 MSPs (n = 7) groups in collagen I, collagen III and the I/III ratio. However, collagen volume fraction (CVF) was significantly lower in infarct zone of animals treated with IGF-1 in comparison to CON and MSPs (22.80 ± 7.69% vs. 48.64 ± 12.09% and 42.90 ± 18.11%, respectively; p = 0.005; One way ANOVA) (Fig. 3A).

After Isolectin B4 immunostaining, a trend towards increased vessel area was observed in infarct and border zones in IGF-1 MSPs group compared to animals belonging to CON and MSPs (Table 5). In remote myocardium, there were significant differences showing more vessel density in IGF-1 MSPs in comparison to the other two groups (p = 0.018; One way ANOVA) (Fig. 3B).

**Table 5:** Vascular area (%) determined by Isolectin B4 immunostaining.

Group	Remote	Infarct	Border
CON	0.82 ± 0.53*	1.10 ± 1.33	0.46 ± 0.44
MSPs	1.46 ± 0.34	1.62 ± 1.74	1.87 ± 2.06
IGF-1 MSPs	4.25 ± 3.22*	2.48 ± 1.96	2.10 ± 1.70

Data presented as mean ± standard deviation. * p < 0.05

## Discussion

This blinded, randomized preclinical study aimed to evaluate whether the administration of encapsulated IGF-1 via the infarct-related coronary artery could improve clinical outcomes. We demonstrated a clear improvement in cardiac function, reflected by better LVEF and ESVi, along with lower CVF and higher vascular density.

In recent years, different subtypes of heart derived progenitor cells have been proven useful in the therapy of ischemic heart disease^16^. However, survival, engraftment, and persistence of transplanted cells or their progeny is limited^17^, so that an important part of these beneficial effects is attributed to cell-secreted paracrine factors^7^ that mediate survival, neovascularization, remodelling and cell proliferation^18,19^ rather than by direct differentiation of engrafted cells.

Indeed, different reports have demonstrated that various cell-liberated substances, such as growth factors, mediate angiogenesis and protect against myocardial ischaemia. In particular, IGF-1 has been shown to possess cardioprotective properties and beneficial effects on the heart^20^. Since the porcine endogenous CSCs have an intact signalling IGF-1/HGF receptor system, the type of growth factor used in our investigation is considered as a good option for MI therapy^14^. Although the effects of administering different growth factors have been analysed in previous experiments^20^, to the best of our knowledge there are no studies that have examined the effect when delivered after its microencapsulation as a way to increase release time in a single intervention.

An important keypoint that has to be considered when assessing the efficacy of a particular therapy is the type of animal model used. To this respect, we have chosen the swine model due to its similarities between the human and the porcine heart, although most of the preclinical studies that have demonstrated significant improvements in cardiac function after treatment have been performed in rodents^14,19^. Undoubtedly, the use of small mammals is useful to establish a proof of concept, the extrapolation of data obtained in these species to human disease, however, is debatable^14^.

In this study we have chosen a temporary balloon occlusion model of the LAD that mimics the clinical situation, in which most of the infarcts are reperfused either spontaneously or therapeutically. In research, the induction of a MI can be achieved by means of different methods. The first one is to create atherosclerosis at the level of the coronary arteries which mimics more accurately the situation of the disease in humans. This technique, however, is rarely used in the preclinical scenario since the induction of the model requires a long period of time.

The second method is based on causing a total or partial blockage of a coronary artery^21,22^ administering drugs (isoproterenol, adriamycin, ergovine) or using either surgical or endovascular techniques, with the latter being clinically more relevant^23^, especially when evaluating treatments for MI.

In the present study, microspheres (either loaded with IGF-1 or not) were delivered into the coronary artery, which represents a widely available administration route in the clinical practice.

One of the greatest advantages of the IC injection is the possibility to combine it with coronary angioplasty, eliminating the need of specific equipment or learning to perform this type of therapeutic procedure^24,25^. Moreover, this minimally invasive approach is able to achieve a homogeneous distribution of the injected therapy^5,16^.

Nevertheless, IC administration carries the risk of causing intimal dissection^26^, microvascular obstruction and even infarction^27–29^, especially when the injected agent is of large size^27,30^.

According to Jong *et al*. the IC administration of encapsulated stem cells of 170 µm in infarcted pigs was safe, which seems to indicate that the IC delivery of the microspheres (16 µm in diameter) injected in the current study is feasible and of low risk^31^.

IC injected products have been reported to disappear very rapidly from the coronary circulation migrating to different locations such as lungs, kidneys and liver^28^. With the aim of improving retention of the delivered therapy, most of the clinical trials that use the IC delivery route have implemented intermittent occlusion for therapy administration^32,33^. This method is intended to yield increased adhesion of the injected substances to the vascular wall, especially when stem cells are used for treatment. Although it is expected that the engraftment is greater in these conditions^34^ experimental studies have demonstrated that retention is similar or even higher when stem cells are administered during uninterrupted coronary blood flow^35^. In accordance with these findings we performed IC infusion through a microcatheter maintaining the normal coronary flow, further diminishing the risk of arrhythmias and vascular wall damage caused by the repeated occlusions^36^. Moreover, the use of biodegradable polymer microspheres of 16 µm in diameter prevents the flow of the encapsulated growth factors through the coronary capillary bed and into the systemic circulation in the first pass, until IGF-1 has been unloaded and the particle degraded. The mentioned features of the used microspheres favour a prolonged and controlled therapy release that could improve results with a single administration procedure.

In order to assess the safety of the IC injection of the microspheres, in our experiment we determined and compared TIMI flow and cTnI values before and after delivery. TIMI flow remained stable in CON group, while it decreased in 4 animals from the MSPs and IGF-1 MSPs groups after the injection. Although a reduction in TIMI flow was evidenced in both groups treated with microspheres, it was not accompanied by increases in cTnI levels that could reveal signs of myocardial damage^37^. Moreover, when we looked at coronary flow at 10 weeks it was again scored as TIMI 3 in almost all animals. Taking all these into account, along with the reportedly safe size^31^ of the injected particles, we concluded that the compromise in flow was temporary and of minimal clinical relevance in these animals.

According to literature cTnI is the biomarker of choice for the diagnosis of myocardial necrosis^38^. In our case, significant differences between groups were detected in the post-reperfusion cTnI values. Taking into account the release kinetics of cTnI- increase starts 2 to 4 hours after acute MI and peak at 24 hours^38^- we assume that these differences could be attributed to small variations in blood collecting times during enzyme elevation period instead of peak. Moreover, it has been described that the value of serum biomarkers such as cTnI can overestimate the necrotic area, especially after reperfusion^39^. To our knowledge, accuracy in determining IS is much higher using further diagnostic tools. Thus, in this preclinical investigation, we used CMR for morphological and functional evaluation of the heart. This imaging technique is widely recommended for the assessment of IS and cardiac function^40–44^ and offers a variety of parameters potentially suited as surrogate end-points in clinical studies^45^. In clinical research the most relevant parameter for assessing heart function is LVEF^42,43^. To this respect, our results reflect an increase over time in this parameter in all groups, significantly so in CON and IGF-1 MSPs. In consonance with our previous experience, LVEF decreases significantly from baseline to pre-injection (post-infarction) and suffers a progressive recovery from that time point to 10 weeks in all groups^6^. Similarly, in the clinical setting it has also been demonstrated that LVEF improves in most patients 1 month after infarction^46^. Myocardial stunning, defined as reversible myocardial dysfunction in regions of normal myocardial perfusion, has been pointed out as the most likely explanation for the increase in LVEF observed in all groups at 10 weeks compared with 2 days post-MI^47^.

In our study, however, at 10 weeks, LVEF was significantly greater in IGF1-MSPs compared to MSPs and CON, which indicates a significant improvement in cardiac function in this group. In comparison with the 3–4% improvement in this parameter obtained with percutaneous coronary intervention^25^, in the IGF-1 MSPs treated animals an 18% increase in LVEF was observed. Likewise, this improvement was greater than the ones documented in other experimental studies based on stem cell therapy^48^ or tissue engineering^49^.

In addition to LVEF, IS and ventricular volume measurements are also considered potentially suited surrogate end-points^45^. In our study the differences in IS were not significant between groups. Nevertheless, a statistically significant decrease over time in this parameter was observed in the three groups. This reduction in the percentage of infarction, including CON and MSPs groups, could be attributed to an overestimation in that parameter in the earliest phases of infarction due to the presence of inflammation, haemorrhage and edema^44,50–52^. Likewise, the decrease in IS at 10 weeks could be explained by the degree of LV wall thinning due to ventricular remodelling resulting in a loss of cardiomyocytes, destruction of the extracellular matrix of the necrotic area and its replacement by a fibrotic scar^53,54^.

TTC staining revealed that ventricular wall thinning was much greater in CON and MSPs groups compared to IGF-1 MSPs. Hence, an apparent decrease in infarcted myocardium was visible in these two groups, as calculated on delayed enhancement CMR images. Conversely, on the TTC stained heart slices obtained from animals belonging to the IGF-1 MSPs group there was little thinning in the infarcted area with presence of a mixture of infarcted and viable tissue. This heterogeneous tissue is represented as grey area on CMR images and thus construed as infarction since these voxels are above the threshold used to define abnormal myocardium^55^. Consequently, no significant differences between groups regarding the percentage of infarction were found, despite the differences in tissue characteristics that were evidenced macroscopically after TTC staining. This dynamic evolution of infarct volume following infarction must be taken into account when using IS as an end-point.

Regarding ventricular volumes, CMR is considered an accurate and highly reproducible technique which is well suited to assess postinfarction remodelling^45^. In the present study, EDVi increased in the three groups over time. ESVi decreased in IGF-1 MSPs group over the 10 weeks period, while it increased slightly in CON and remained stable in MSPs. These results, especially the one´s found in the IGF-1 MSPs group, agree with data described in literature that indicate that EDVi increases, ESVi decreases and compensatory hypertrophy of the remote myocardium are common findings after MI in order to preserve stroke volume and LVEF^44^.

Microscopically, post-mortem analysis (H/E and MT staining) revealed no anatomopathological differences between groups neither in remote, nor in infarct and border zones. Macroscopically, however, IGF-1 treated animals exhibited viable tissue regions within the fibrous scar tissue as mentioned before.

Before assessing the effectiveness attributable to a particular treatment it is necessary to have a broad knowledge of the pathological consequences caused by MI. The colour and the appearance of the damaged myocardial tissue varies depending on the time elapsed after the infarction. Thus, after 10 weeks a firm and white scar that is generally thinner than the surrounding myocardium would be present^56^. Microscopically a fibrous scar is established which is characterized by the presence of compact and dense collagen deposits^57^. During normal wound healing, initially type III collagen fibers, contributing to maintenance of normal heart shape and stiffness of the myocardium, are constructed throughout the infarct zone. Later, these fibers are normally replaced by type I collagen, exhibiting high stiffness and thus allowing ventricular remodelling^58^.

Myocardial fibrosis is characterized by the increase in the percentage of total myocardial tissue occupied by collagen fibers, denoted as CVF. In our study we observed that heart samples belonging to IGF-1 MSPs group exhibited a significantly lower CVF (and ultimately less fibrosis), which has been associated in other studies with improved LV diastolic dysfunction and decreased LV stiffness^59^ and was consistent with the presence of viable areas within the infarcted myocardial tissue detected after TTC staining.

The results of our study demonstrated that the IC administration of IGF-1 loaded microspheres at the assayed dose enhanced angiogenesis after MI, although, this angiogenic effect reached statistical significance only in remote areas of the myocardium. It is now widely recognized that the remote myocardium is a dynamic environment that evolves in parallel to changes occurring in the infarct area. For example, CMR has revealed changes in this area, such as an increase in T1 values from 4 days to 3 months after infarction^60^, changes that were, at least in that study, related to the severity of infarction. In a recent paper studying microvascular changes using 3D fully automated image analysis at different timepoints after infarction, a clear decrease in capillary density, along with increased intercapillary distance were found in the infarct core and also in the remote myocardium, although remote changes were milder. This can lead to impaired oxygen diffusion and subsequent decrease in metabolic capacity and contractile force^61^. The severity of microvascular disfunction has been associated to the progression of hibernating towards necrotic myocardium, so that functional microvasculature is necessary to avoid cardiomyocyte dead leading to infarct expansion^62^. Since cardiomyocyte growth and survival, along with contractile capacity, depend upon microvasculature, it follows that enhanced microvascular density can lead to improved cardiac contractility. Similarly to ours, prior studies have reported increases in capillary density in the remote myocardium in absence of significant arteriogenesis after infusion of CDCs and MSCs^63^. Interestingly, these increases matched with increased nuclei density, so that the rate of capillary to myocyte was maintained. While no increases in perfusion could be evidenced, probably due to the negligible contribution of the capillaries to the actual vascular resistance, function was improved in both cases compared to vehicle treated animals, as we have seen in our IGF-1 treated subjects. The authors postulated that this may facilitate oxygen exchange and transport at the cellular level, which in turn could have a beneficial effect on contractility and therefore contribute to the improved function. Moreover, endothelial cell to cardiomyocyte cross-talk has been reported to control cardiomyocyte contractility^62^. In our case, the increased capillary density could be key player in improving cardiac function, and thus may be responsible for the improved LVEF in absence of differences in IS in the three groups.

Although the swine model of acute MI is the most attractive one for preclinical studies^14,64^ of myocardial regeneration it is not exempt from certain limitations. On the one hand, in our case we have chosen healthy animals without cardiovascular risk factors and whose coronary circulation is not compromised by conditions such as atherosclerosis^65^. On the other hand, in this kind of animal model, the beginning and the end of the coronary occlusion period were clearly defined. This situation is completely different in the human patient^66^. Moreover, we used juvenile pigs that experienced an increase in size throughout the study. Growing hearts do not exactly reflect the process of remodelling that occurs in adult patients after an infarction, which can cause confusion when assessing the effectiveness of a particular treatment. Likewise, the rapid growth of these animals complicates their long-term follow-up, so in recent years the use of miniature breed pigs has been proposed^67^. In the present study, with the aim of reducing the influence of weight gain on our interpretation of the results, as previously reported by others^16^, ventricular volumes have been indexed to Body Surface Area.

In this study, local and circulating levels of IGF-1 were not determined, neither before nor after the IC injection. Moreover, *in vivo* release kinetics of IGF-1 were not defined and the changes in number and activity of local progenitor cells have not been monitored in this experimental study, and therefore have to be mentioned as further limitations.

Although we carried out comparisons between IGF-1 MSPs, CON and MSPs groups, an additional group of animals comparing IGF-1 MSPs with IGF-1 alone would have been useful to clarify if microencapsulation of this therapeutic agent could be able to improve results.

Finally, antiarrhythmic medication was used before model creation in order to reduce mortality rate during the study. This fact could have masked any arrhythmogenic effects during IC injection.

In conclusion, the IC administration of IGF-1 loaded MSPs at the assayed concentration could slightly impair coronary flow on short term although it is not reflected in cTnI values and therefore caution must be exerted when considering clinical translation. From an efficacy point of view, however, the IC infusion of 5 × 10^6^ IGF-1 loaded MSPs in an experimental acute MI model was successful in improving cardiac function, as seen by an increase in LVEF. Although no significant differences between groups were observed in indexed ventricular volumes and vascular density in infarct or border zones, the reduced CVF in animals treated with IGF-1 loaded MSPs indicates a limitation of myocardial fibrosis that, together with the improved LVEF, could be relevant in clinical practice.

## Supplementary information

Supplementary information.

## Data Availability

The datasets generated and analysed during the current study are available from the corresponding author on reasonable request.
