# Supplementary material for: Microencapsulated Insulin-Like Growth Factor-1 therapy improves cardiac function and reduces fibrosis in a porcine acute myocardial infarction model
Source: Sci Rep. 2020 Apr 28;10:7166. doi: 10.1038/s41598-020-64097-y (PMC7188803; doi:10.1038/s41598-020-64097-y)
Supplement: Supplementary file 1 — Supplementary information. [file 41598_2020_64097_MOESM1_ESM.pdf]

Supplementary Figures

Microencapsulated Insulin-Like Growth Factor-1 therapy improves cardiac function and reduces fibrosis in a porcine acute myocardial infarction model

Claudia Báez-Díaz[1,2,*], Virginia Blanco-Blázquez[1,2], Francisco-Miguel Sánchez-Margallo[1,2,*], Antonio Bayes-Genis[2,3], Irene González[1], Ana Abad[1], Rob Steendam[4], Okke Franssen[5], Itziar Palacios[6], Belén Sánchez[6], Carolina Gálvez-Montón[2, 3], Verónica Crisóstomo[1,2].

1.- Jesús Usón Minimally Invasive Surgery Centre, Cáceres, Spain.

2.- CIBERCV, Madrid, Spain

3.- ICREC (Heart Failure and Cardiac Regeneration) Research Programme, Health Sciences Research Institute Germans Trias i Pujol (IGTP), Badalona, Barcelona, Spain

4.- Innocore Pharmaceuticals, Groningen, The Netherlands.

5.- Nanomi BV, Oldenzaal, The Netherlands.

6.- Tigenix, Madrid, Spain

* Corresponding authors

E-mail: cbaez@ccmijesususon.com (C.B.D.); msanchez@ccmijesususon.com (F.M.S.M.)

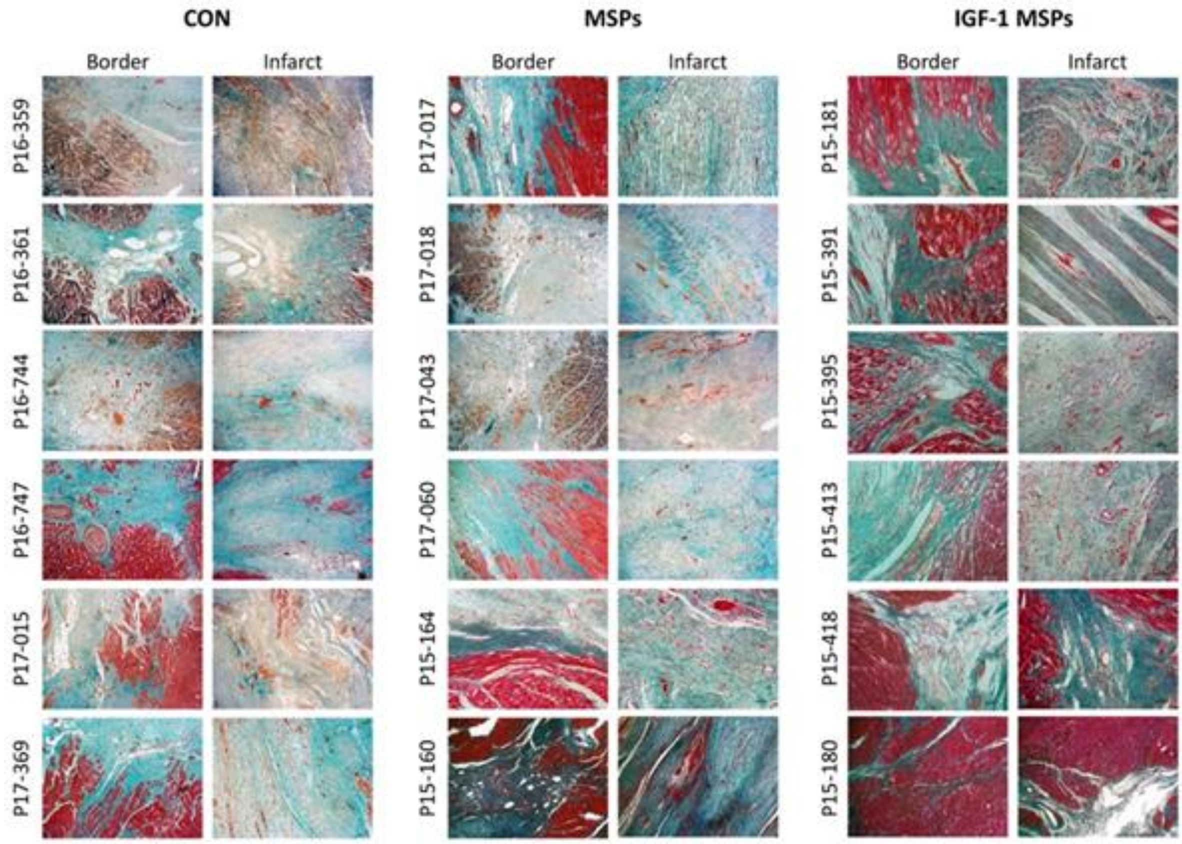

Supplementary Figure S1. Representative histological images of complete MT staining from CON (n=6), MSPs (n=6), and IGF-1 MSPs (n=6) groups.

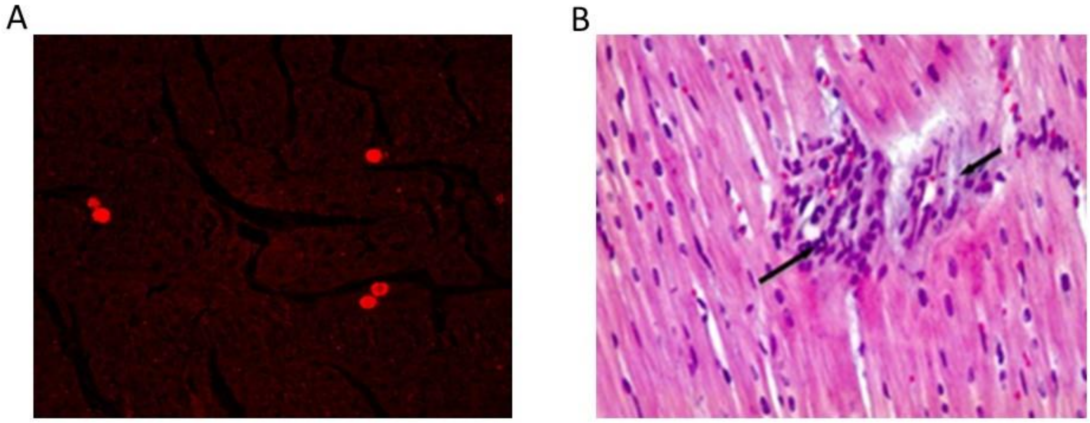

Supplementary Figure S2. Representative images of red labeled MSPs distribution in healthy pigs after IC injection. A) Fluorescence microscopy detection of the microspheres in porcine myocardium. B) H/E staining shows a mild inflammation around the MSPs (arrows). Scale bar = 50 μm.
